# Functional Outcomes and Activity Levels in Patients After Internal Hemipelvectomy for Primary Sarcoma Involving the Bony Pelvis

**DOI:** 10.3390/diagnostics15121452

**Published:** 2025-06-06

**Authors:** Burkhard Lehner, Jakob Bollmann, Andreas Geisbüsch, Nicholas Andreas Beckmann

**Affiliations:** 1 Department of Orthopaedics, Heidelberg University Hospital, 69118 Heidelberg, Germany; burkhard.lehner@med.uni-heidelberg.de (B.L.); andreas.geisbuesch@med.uni-heidelberg.de (A.G.); nicholas.beckmann@med.uni-heidelberg.de (N.A.B.); 2 German Bone Tumor Working Group, 4031 Basel, Switzerland

**Keywords:** internal hemipelvectomy, musculoskeletal sarcoma, pelvis, functional outcomes, sport activity levels

## Abstract

**Background:** Internal hemipelvectomies are rare procedures for primary musculoskeletal sarcomas of the bony pelvis. There is a sparse amount of data on functional outcomes and activity levels in postoperative patients. The aim of this study was to investigate functional outcomes, including sport activity levels, and the impact of tumor grade, resection margins, adjuvant therapies, pelvic reconstruction, and patient age at the time of surgery. **Methods:** Patients who underwent internal hemipelvectomy at our clinic between 1995 and 2019, with a minimum follow-up of 12 months, were assessed using the Musculoskeletal Tumor Society Score (MSTS), the Toronto Extremity Salvage Score (TESS), the Oxford Hip Score (OHS), and the University of Los Angeles Activity Scale (UCLA AS). **Results:** Our cross-sectional study included 29 patients (14 male, 15 female; 15 with chondrosarcoma, 8 with Ewing’s sarcoma, 2 with osteosarcoma, 2 with chordoma, and 2 with other sarcomas) with a median follow-up of 8.7 years (range: 12 months to 25.4 years; interquartile range (IQR): 13.1 years). The median MSTS was 16 (range: 1–30; IQR: 9), median TESS was 75.8% (range: 12.9–100%; IQR: 31.7%), median OHS was 35 (range: 10–48; IQR: 16), and median UCLA AS was 5 (range: 1–9; IQR: 3). Tumor grade, resection margins, chemotherapy, radiation therapy, and pelvic reconstruction had no significant effect on functional outcomes. Patient age at the time of surgery had a statistically significant effect on all measured outcome parameters, although all parameters exhibited a wide range and large IQR, likely reflecting the small, heterogeneous patient cohort. **Conclusions:** Surviving patients who underwent internal hemipelvectomy for primary musculoskeletal sarcomas of the pelvic bone demonstrated overall moderate to good functional outcomes and moderate sport activity levels.

## 1. Introduction

Primary musculoskeletal tumors that arise in or intimately involve bone are very rare compared to other primary neoplasms, and benign tumors are significantly more common than malignant ones. The estimated incidence of primary sarcomas affecting bone is approximately 1 per 100,000 persons per year, accounting for only about 0.2% of all malignant neoplasms [1,2,3]. Most primary malignancies affecting bone occur in the long bones and only rarely involve the pelvic bones.

Most pelvic musculoskeletal malignancies grow silently and remain asymptomatic until they reach a considerable size. Treatment is interdisciplinary, multimodal, and depends on the specific tumor type, location, and extent. When surgery is considered potentially curative, the initial approach is complete surgical resection with the goal of tumor-free margins. However, in many cases, neoadjuvant or adjuvant chemotherapy and/or radiation therapy is required.

**Figure 1:**
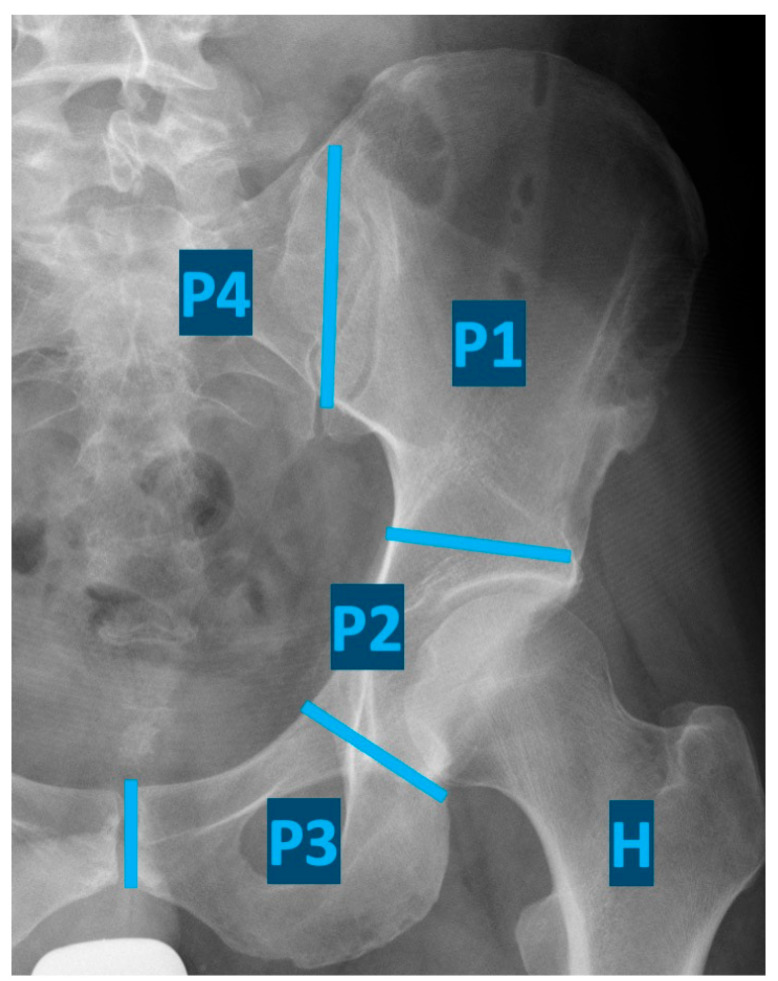
Classification of internal hemipelvectomies according to Enneking (resection of the iliac wing (P1), periacetabular region (P2), pubis and ischii (P3), sacrum (P4), and proximal femur (H)).

Potentially curative surgical intervention for musculoskeletal sarcomas involving the pelvic bones requires an internal hemipelvectomy, a highly complex procedure that must be individualized based on patient age, comorbidities, and tumor characteristics, including size, location, and histologic type. The extent of resection is classified according to the Enneking classification (Figure 1) [4,5]. In some patients, tumor infiltration of the hip joint and periarticular tissues necessitates the resection of the proximal femur. Pelvic reconstruction is likewise tailored to the individual patient, depending on patient characteristics and the extent of resection [6].

Little is known about functional outcomes and activity levels in patients who have undergone internal hemipelvectomy. Most studies include small patient cohorts, and reported mean functional outcomes vary widely [7,8,9,10,11,12]. To the best of our knowledge, no data are available on sports activity levels in postoperative patients. The aim of this study was to investigate functional outcomes and activity levels in patients who have undergone internal hemipelvectomy, using patient-reported outcome measures (PROMs). Additionally, we sought to assess the influence of tumor grade, resection margins, adjuvant radiation and chemotherapy, pelvic reconstruction, and patient age at the time of surgery on functional outcomes.

## 2. Materials and Methods

This cross-sectional study included patients who underwent internal hemipelvectomy at the Orthopedic Hospital Heidelberg between 1995 and 2019 for a variety of primary malignant and potentially curative musculoskeletal tumors of the bony pelvis. Included patients had a follow-up period of at least 12 months and were at least 12 years old at the time of the follow-up assessment.

Exclusions were made for patients unable to provide informed consent due to language barriers, those living abroad, and those unwilling to give consent.

**Figure 2:**
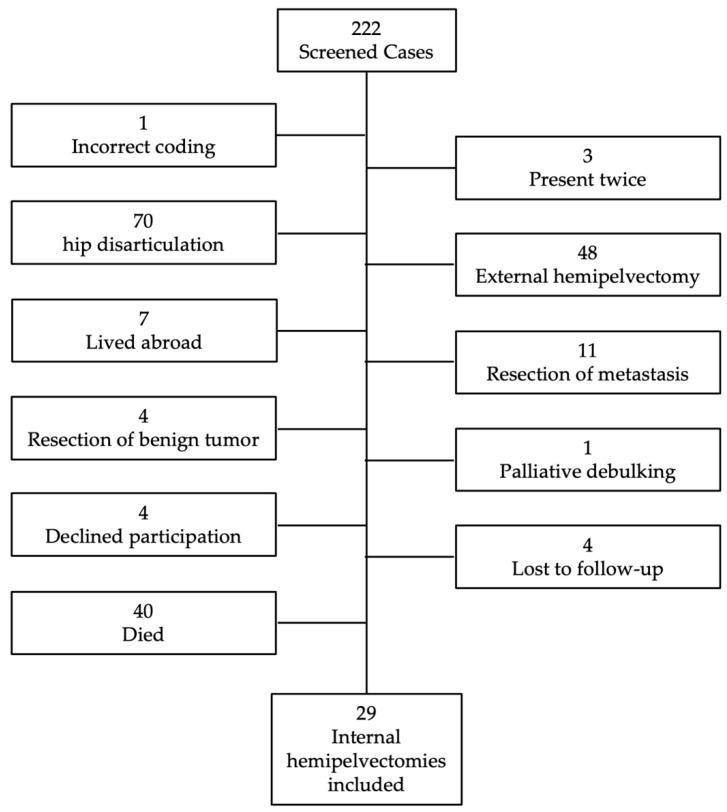
Flow chart showing the excluded patients.

Specific surgical procedures are individually coded in our hospital database, and our search for the coded resection of tumors involving the hip and pelvic bones yielded 222 patients treated during the specified time frame. Of these, one was incorrectly coded, three were duplicated in the system, 70 underwent hip disarticulation, 48 received an external hemipelvectomy, 7 resided abroad, 11 had resections for tumor metastases, 4 had resections of benign tumors, and 1 received a local palliative debulking surgery. Of the remaining 77 patients, 4 declined participation, and 4 were lost to follow-up, leaving 69 patients. Of these, 40 were deceased at the time of the study (median time to death: 2.1 years; range: 0.3 to 20.5 years). Therefore, 29 patients remained and were included in this study (Figure 2).

After obtaining informed consent, functional outcomes and sports activity levels were assessed using patient-reported outcome measures (PROMs) collected during outpatient hospital visits or sent by mail.

For a general functional evaluation, we administered the Musculoskeletal Tumor Society Score (MSTS) for the lower extremity and the Toronto Extremity Salvage Score (TESS), also for the lower extremity [13,14,15]. We also assessed the Oxford Hip Score (OHS), originally intended for patients who underwent total hip arthroplasty [16,17]. Internal hemipelvectomies preserve the lower limb; therefore, the OHS can be used to assess the function of the hip joint postoperatively. The OHS can be divided into two subscales: one for pain and one for function [18].

To assess postoperative sports activity levels, the University of California Los Angeles Activity Scale (UCLA AS) was used. Patients rate their physical activity from 1 (completely inactive and dependent on others) to 10 (regular participation in impact sports) [19].

Oncologic and demographic data were obtained from the hospital database. The data were pseudonymized, stored in an Excel file, and transferred to SPSS (Version 28, IBM Corporation, Armonk, NY, USA) for statistical analysis. For metric variables, the minimum, median, maximum, standard deviation (SD), and interquartile range (IQR) were calculated in a descriptive data analysis. To assess whether grading, resection margins, chemotherapy, radiation therapy, or pelvic reconstruction after tumor resection influenced functional outcomes, the PROMs were compared using the Mann–Whitney-U test. The correlation between age at the time of surgery and functional outcome was determined using Pearson’s correlation coefficient. A *p*-value of < 0.05 was considered statistically significant.

## 3. Results

Our 29 patients (14 male, 15 female) had a mean age of 38 years at the time of surgery (range: 8–70 years). The cohort included 15 (52%) patients treated for chondrosarcoma, 8 (27%) for Ewing sarcoma, 2 (7%) for osteosarcoma, 2 (7%) for chordoma, 1 (3%) for undifferentiated pleomorphic sarcoma, and 1 (3%) for alveolar soft tissue sarcoma. The area of the pelvis most commonly affected was the ilium (76%). Other affected areas included the pubis (10%), sacrum (7%), femur (3%), and gluteal region (3%). In most cases, the tumor was high grade (grade 2: 27%, grade 3: 59%), while 10% were classified as low grade (grade 1), and one tumor could not be graded.

An overview of the surgical resections according to the Enneking classification is provided in Table 1.

Clear resection margins (R0 resection) were achieved in 21 (71%) patients. Five patients had resection margins showing the presence of tumor cells on histological examination (R1 resection). In three (10%) patients, the resection margins could not be clearly defined (RX resection). Chemotherapy alone was administered to 5 (17%) patients, radiation therapy alone to 3 (10%) patients, both chemotherapy and radiation therapy to 9 (31%) patients, and 12 (41%) patients received neither chemotherapy nor radiation therapy.

Following tumor resection, 11 (38%) patients underwent individualized pelvic reconstruction. Among these, three received a hemi-endoprosthesis combined with a bipolar head and fixation using an attachment tube to the residual sacral bone, two patients received a pedestal cup combined with a standard hip prosthesis stem, and one patient received a pedicle screw rod-system coated with bone cement. An additional five patients underwent biological reconstruction: three patients received an autologous fibula fixed with screws or plates, one patient had resected bone irradiated extracorporeally, then reimplanted and fixed with plates, and one patient received an allogenic femoral head fixed to the remaining acetabulum to provide support for the residual proximal femur.

At least one revision surgery was required in 10 (34%) patients. The first revision surgery was performed due to infection in six patients and inadequate wound healing in four patients.

Postoperative tumor progression occurred in seven (24%) patients: one patient experienced local recurrence (resected with clear margins and was tumor-free at the latest follow-up), three patients developed pulmonary metastases (one received whole-lung radiation shortly after the initial surgery, one underwent lobectomy (both were tumor-free at the latest follow-up), and one patient had slowly progressing bilateral pulmonary metastases), and three patients developed both local recurrence and pulmonary metastases (all were receiving palliative care at the latest follow-up).

**Figure 3:**
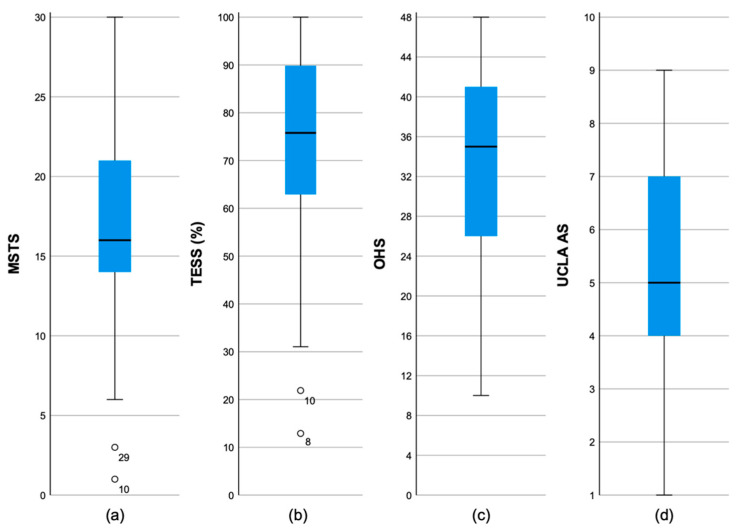
Boxplots of the functional PROMs: (**a**) Musculoskeletal Tumor Society Score; (**b**) Toronto Extremity Salvage Score; (**c**) Oxford Hip Score; (**d**) University of California Activity Score.

The median follow-up of the 29 patients was 8.7 years (range: 1.1–25.4 years; SD: 7.4 years). At the latest follow-up, the median MSTS was 16 (53.3%) (range: 1 to 30; interquartile range (IQR): 9; SD: 6.9). The median TESS was 75.8% (range: 12.9–100%; IQR: 31.7%; SD: 23.4%). The median OHS was 35 (73%) (range: 10–48; IQR: 16; SD: 11.1) with a median pain subscale score of 16 (67%) and a median function subscale score of 19 (79%). The median UCLA AS was 5 (range: 1–9; IQR: 3; SD: 2.2) (Figure 3).

Tumor grade (low grade (G1) vs. high grade (G2/G3)), resection margins (RO vs. R1/RX), chemotherapy (+/−), radiation therapy (+/−), or pelvic reconstruction (+/−) had no significant influence on functional outcomes at follow-up (Table 2).

**Figure 4:**
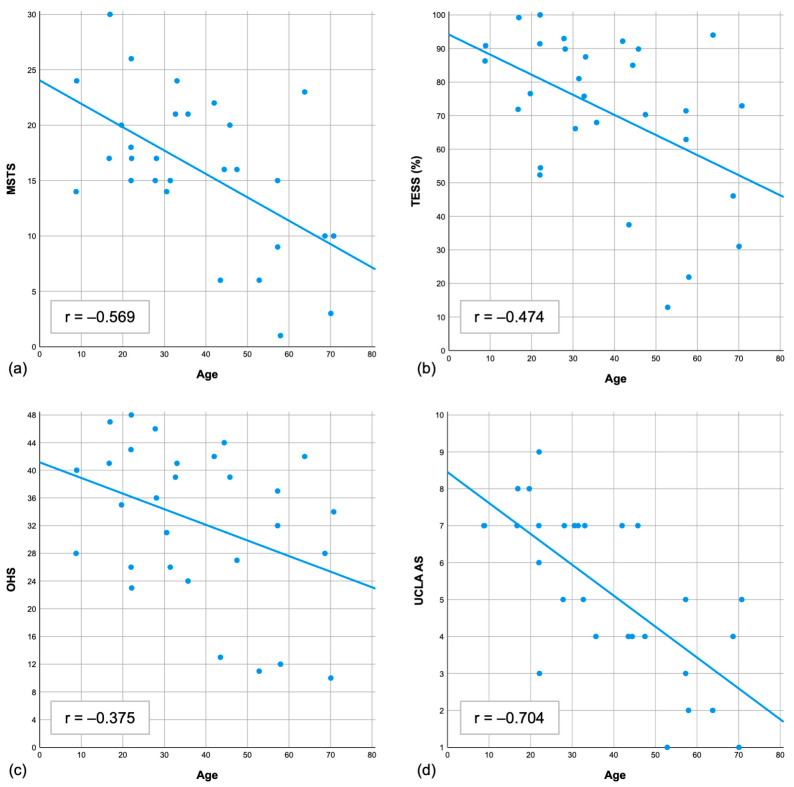
Scatter plots showing the correlation between age at the time of surgery and the functional PROMs. Each plot demonstrates a clear linear correlation: (**a**) Musculoskeletal Tumor Society score; (**b**) Toronto Extremity Salvage Score; (**c**) Oxford Hip Score; (**d**) University of California Activity Score.

Patient age at the time of surgery had a significant influence on all four functional outcome parameters. Younger patients at the time of surgery showed better functional outcomes (MSTS: r = −0.569, *p* = 0.001; TESS: r = −0.474, *p* = 0.009; OHS: r = −0.375, *p* = 0.045; UCLA AS: r = −0.704, *p* < 0.001) (Figure 4).

When dividing the cohort into two groups (patients younger than 40 years (*n* = 16) and patients older than 40 years (*n* = 13) at the time of surgery), these trends were confirmed by analysis using the Mann–Whitney-U test (younger than 40 years vs. older than 40 years: median MSTS: 18 vs. 10, *p* = 0.011; TESS: 84% vs. 70%, *p* = 0.057; OHS: 38 vs. 32, *p* = 0.195; UCLA AS: 7 vs. 4, *p* < 0.001) (Table 3).

## 4. Discussion

Internal hemipelvectomies are rarely performed; therefore, no prospective studies exist, and the few retrospective follow-up studies include only small patient cohorts [20]. The limited studies that have assessed functional outcomes also involve very small cohorts with highly variable follow-up periods, ranging from a few months to 30 years [7,21,22]. Very few studies exist that have evaluated the MSTS in cohorts of more than 25 patients following internal hemipelvectomy for sarcomas of the bony pelvis [23,24,25,26,27]. One study assessed the MSTS in 28 patients after internal hemipelvectomy for bone sarcoma involving the sacro-iliac joint, reporting a mean MSTS of 17 points at a mean follow-up of 64 months [24]. A second study examined 45 patients after resection of a periacetabular chondrosarcoma, with an MSTS of 20.5 points (68.3%) at a mean follow-up of 37 months [23]. A multicenter study reported a mean MSTS of 53% (16 points) at a median follow-up of 17 months after hip transposition following periacetabular resection of a malignant pelvic tumor [27]. A study of 96 patients treated with three-dimensionally printed custom hemipelvic endoprostheses for primary pelvic sarcoma reported a mean MSTS of 23.8 points at a mean follow-up of 48 months [25]. The most recent study reported a mean MSTS of 18 points in patients who received a three-dimensionally printed prosthesis for acetabular reconstruction following resection of a primary or secondary pelvic malignancy [26]. These MSTS values are comparable to our median MSTS of 16 points, despite our considerably longer median follow-up period of nearly 9 years. However, comparisons among these studies are of limited clinical value due to differences in tumor types, grades and locations, treatment protocols, and patient demographics.

Published studies assessing the TESS following internal hemipelvectomy have reported mean TESSs ranging from 58 to 76%, slightly higher than corresponding MSTSs [11,12,28,29,30]. However, patient cohorts were small in all but one study. In our study, the median TESS was 75.8%, which aligns with prior findings, though comparisons are limited by the small sample sizes. These TESS results suggest that long-term survivors can achieve a good level of functional activity.

Our study also utilized the validated OHS scale to assess hip-specific functional activity. The median OHS in our cohort was 35 (73%) points, which is comparable to our TESS. The OHS includes two subscales: pain and function. Our median pain subscale score was 67%, while the median function subscale score was 79%, indicating that chronic pain presents a greater limitation than functional impairment of the hip. Notably, our median OHS was considerably lower than the 85 to 90% typically achieved by patients undergoing total hip arthroplasty (THA) for primary osteoarthritis [31,32].

All three functional PROMs used in our study showed a wide range of scores, reflecting substantial variability in functional ability among individual patients.

We found no existing data reporting sport activity levels in patients after internal hemipelvectomy. Our median UCLA AS score was 5 (indicates “some participation in moderate activities such as swimming or able to do unlimited housework and shopping” [19]), with scores ranging from 1 to 9. Our mean score aligns with previously reported scores of 5 to 6 in patients treated surgically for musculoskeletal tumors of the lower extremity [33,34,35,36]. The broad range (1 to 9) in our cohort underscores the significant variability in sport ability levels among individuals.

Our results show that, despite considerable individual variation, patients achieved moderate to good functional outcomes on average, suggesting a high degree of functional independence in daily life and successful social reintegration. A comparison with other studies on functional outcomes after internal hemipelvectomy indicates that our patients achieved comparable results.

None of our functional PROM results showed a clinically significant effect of tumor grade, resection margins, adjuvant radiation or chemotherapy, or pelvic reconstruction on functional or sport activity outcomes. This may be due to our small sample size and the heterogeneity of tumor types, tumor locations, treatment approaches, and patient characteristics. As the influence of reconstruction, chemotherapy, or radiation therapy on functional outcomes and sport activity levels remains uncertain, surgical planning must continue to be tailored to the individual patient. Different reconstruction options (e.g., reconstruction of an articulating hip joint or leaving a Girdlestone situation) may have varying effects on the different domains of functional PROMs, such as pain or walking ability. Further studies investigating the specific impact of reconstruction on functional outcomes are necessary to determine which type of reconstruction is most appropriate for each individual patient.

In our study, age at the time of surgery was the only factor significantly associated with functional and sport activity outcomes. We found a clear correlation (Figure 4), with younger patients demonstrating significantly better functional results in all outcome scales. This is not unexpected, as increasing age is generally linked to higher rates of comorbidity, including degenerative joint disease.

Our study has several limitations. Primary musculoskeletal tumors of the bony pelvis are very rare, leading to a small and heterogeneous patient cohort with varying ages, comorbidities, tumor types and locations, and treatment modalities (e.g., different reconstruction methods), resulting in a selection bias. This likely contributes to the wide ranges and large standard deviations observed in our PROM results. In particular, the absence of significant differences in the subgroup analyses (tumor grade, resection margin, chemotherapy, radiation therapy, and pelvic reconstruction) is likely due to the small sample size. Additionally, the extended follow-up period could have influenced outcomes, inherent to the retrospective cross-sectional study design. However, to avoid false negative results during early postoperative phases, we included only patients with a minimum follow-up of 12 months. Of the 69 eligible internal hemipelvectomy patients, 40 had already died at the time of data collection. This may have excluded poorer outcomes and positively skewed the functional results. A standardized collection of PROMs during postoperative follow-up examinations would help capture the functional outcomes of all patients. Some patients required revision surgery during follow-up, introducing further selection bias, and the impact of such revisions on functional outcomes remains unclear. Due to the limited sample size, we were unable to analyze the impact of specific effects of tumor type, location, and specific treatment modalities on functional outcomes. Our use of patient-reported outcome measures (PROMs) means that the results are subjective and may be influenced by patients’ mood or other health and emotional factors. Objective assessments such as the Timed Up and Go test or the six-minute walk test could complement PROMs and provide more robust functional evaluations. Nevertheless, based on our study results, patients requiring an internal hemipelvectomy due to a primary malignant musculoskeletal tumor in the pelvis can now be better informed about the expected postoperative functional outcomes and sport activity levels.

## 5. Conclusions

Surviving patients who underwent internal hemipelvectomy for primary musculoskeletal sarcomas of the pelvic bone demonstrated overall moderate to good functional outcomes and retained the ability to perform daily activities, although some experienced persistent chronic pain. Sport activity levels were moderate, with considerable variability across patients. Age at the time of surgery was the only factor that significantly impacted both functional and sport activity outcomes, though it remains unclear to what extent comorbidities in older patients and the small, heterogeneous cohort contributed to this finding. Larger studies incorporating objective functional tests may help clarify factors influencing functional and sport outcomes following internal hemipelvectomy.

## Figures and Tables

**Table 1:** Frequency of resection types.

Type of Resection	Frequency (%)
P1	9 (31)
P1 + P4	5 (17)
P1 + P2	3 (10)
P4	2 (7)
P1 + P2 + P3	2 (7)
P2 + P3	2 (7)
P1 + P2 + P3 + P4 + H	2 (7)
P1 + P2 + P4	2 (7)
P1 + P2 + H	1 (3)
P1 + P2 + P3 + H	1 (3)

**Table 2:** Medians and differences in the used PROMs with respect to tumor grade, resections margins, chemotherapy, radiation therapy, and reconstruction.

Functional Outcome	Variables (Median)		Difference	*p*-Value
	Low grade (G1) (*n* = 3)	High grade (G2/G3) (*n* = 25)		
MSTS	15	16	+1	0.742
TESS	76%	77%	+1%	0.720
OHS	32	36	+4	0.607
UCLA AS	5	6	+1	0.760
	R0 (*n* = 21)	R1/RX (*n* = 8)		
MSTS	16	17	+1	0.839
TESS	76%	75%	−1%	0.822
OHS	36	35	−1	0.658
UCLA AS	5	6	+1	0.468
	Chemotherapy (*n* = 14)	No chemotherapy (*n* = 15)		
MSTS	17	16	−1	0.872
TESS	79%	76%	−3%	0.923
OHS	32	35	+3	0.723
UCLA AS	7	5	−2	0.849
	Radiation therapy (*n* = 12)	No radiation therapy (*n* = 17)		
MSTS	17	15	−2	0.562
TESS	79%	76%	−3%	0.534
OHS	34	35	+1	0.819
UCLA AS	7	5	−2	0.648
	Reconstruction (*n* = 11)	No reconstruction (*n* = 18)		
MSTS	15	18	+3	0.155
TESS	66%	76%	+10%	0.381
OHS	28	38	+10	0.143
UCLA AS	6	5	−1	0.901

**Table 3:** Median values, interquartile range (IQR), and *p*-values for the four different PROMs, comparing patients younger and older than 40 years of age at the time of operation.

PROMS	Age < 40 (Median (IQR))	Age > 40 (Median (IQR))	*p*-Value
MSTS	18 (8)	10 (12)	0.011
TESS	84% (22%)	70% (53%)	0.057
OHS	38 (16)	32 (28)	0.195
UCLA	7 (2)	4 (3)	<0.001

## Data Availability

All data are available upon request.
